# Liquid biopsies in cancer

**DOI:** 10.1186/s43556-025-00257-8

**Published:** 2025-03-20

**Authors:** Hang Yin, Manjie Zhang, Yu Zhang, Xuebing Zhang, Xia Zhang, Bin Zhang

**Affiliations:** 1 The First Affiliated Hospital of Dalian Medical University, Dalian, 116000 China; 2 Dalian Medical University, Dalian, 116000 China; 3 Dalian Fifth People’s Hospital, Dalian, 116000 China

**Keywords:** Circulating tumor DNA, Liquid biopsy, Circulating tumor cell, Minimal residual disease, Prognosis, Personalized medicine

## Abstract

Cancer ranks among the most lethal diseases worldwide. Tissue biopsy is currently the primary method for the diagnosis and biological analysis of various solid tumors. However, this method has some disadvantages related to insufficient tissue specimen collection and intratumoral heterogeneity. Liquid biopsy is a noninvasive approach for identifying cancer-related biomarkers in peripheral blood, which allows for repetitive sampling across multiple time points. In the field of liquid biopsy, representative biomarkers include circulating tumor cells (CTCs), circulating tumor DNA (ctDNA), and exosomes. Many studies have evaluated the prognostic and predictive roles of CTCs and ctDNA in various solid tumors. Although these studies have limitations, the results of most studies appear to consistently demonstrate the correlations of high CTC counts and ctDNA mutations with lower survival rates in cancer patients. Similarly, a reduction in CTC counts throughout therapy may be a potential prognostic indicator related to treatment response in advanced cancer patients. Moreover, the biochemical characteristics of CTCs and ctDNA can provide information about tumor biology as well as resistance mechanisms against targeted therapy. This review discusses the current clinical applications of liquid biopsy in cancer patients, emphasizing its possible utility in outcome prediction and treatment decision-making.

## Introduction

Cancer is a primary factor contributing to global mortality; 20 million people were diagnosed with cancer, and millions died from the disease in 2022. According to a report by Freddie Bray et al. in 2024, cancer was the primary or secondary cause of mortality in 112 out of 183 nations and was the third leading cause of mortality in 23 additional countries [1].

**Fig. 1:**
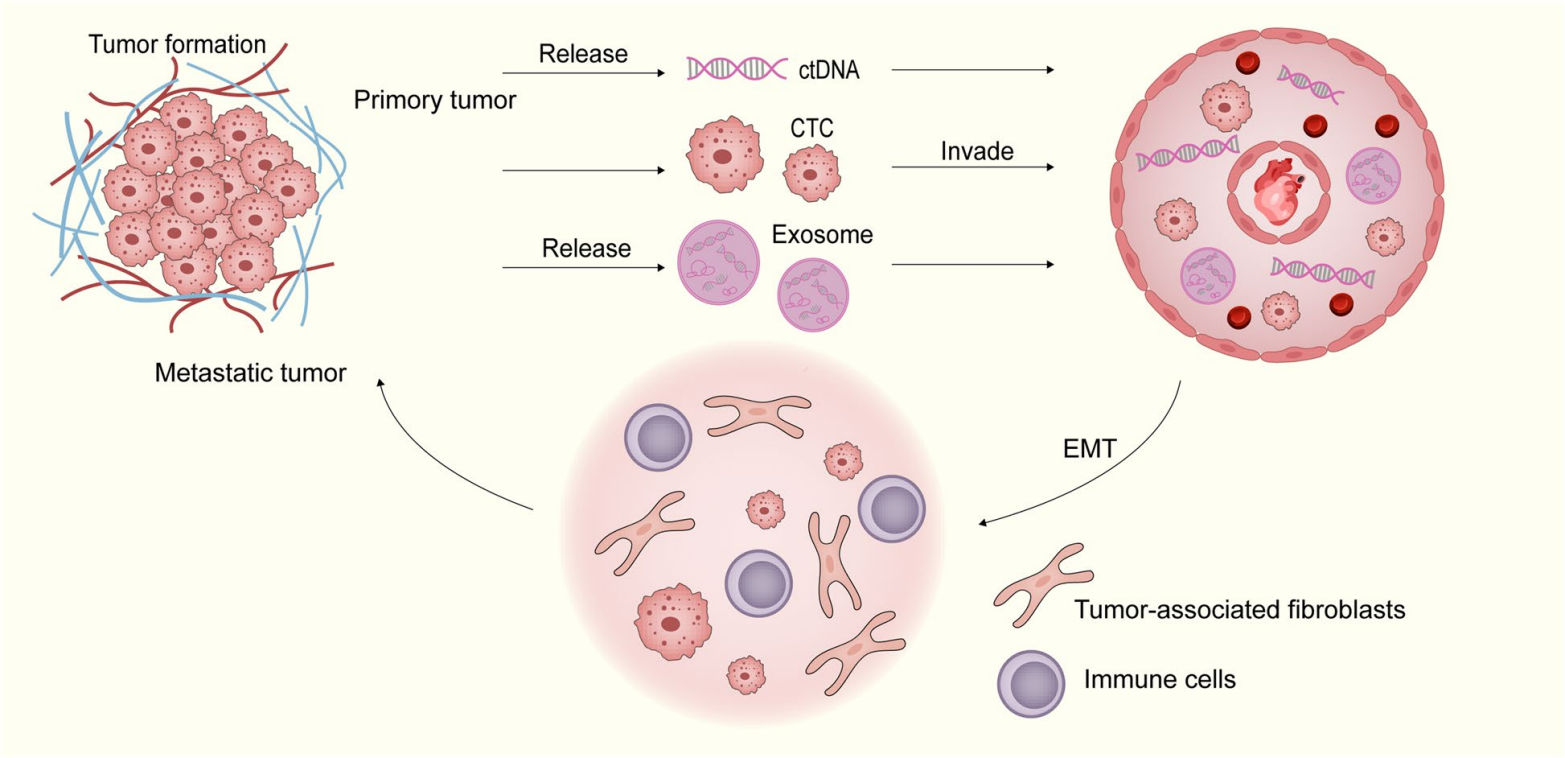
The primary tumor site, due to microenvironmental conditions such as hypoxia, triggers epithelial–mesenchymal transition (EMT). Then, the tumor-related substances are released into the blood. Circulating tumor cells (CTCs) have the capacity to create clusters either among themselves or with other immune cells or cancer-associated fibroblasts. This clustering enhances their metastatic potential, proliferation capability, stemness, and ability to evade the immune system. Ultimately, tumor cells establish a premetastatic niche and colonize a distant site following extravasation

As cancer represents a significant global public health issue, the early detection of cancer and the development of personalized treatment plans are important factors in improving the outcomes and survival times of cancer patients [2]. Currently, tissue biopsy remains the gold standard for tumor diagnosis. However, there are challenges related to difficulty in sample collection, potential harm to patients, and the inability to continuously monitor changes and progression of the disease. Additionally, most tumors have an insidious onset, making accurate detection in the early stages via tissue biopsy challenging [3]. A new technique called liquid biopsy has been developed to detect tumors and their features and monitor progression on the basis of analyzing biomarkers such as circulating tumor cells (CTCs) and circulating tumor DNA (ctDNA) in bodily fluid samples. Owing to microenvironmental conditions such as hypoxia, epithelial‒mesenchymal transition (EMT) is triggered at the primary tumor site. EMT promotes the detachment of tumor cells, allowing them to infiltrate the bloodstream. This leads to the presence of various tumor-related substances in the circulatory system (Fig. 1). These biomarkers provide information about genetic variations in tumors, tumor burden, and other crucial details, helping to guide tumor treatment decision-making and monitor disease progression. The primary advantages of liquid biopsy are its potential application in screening studies and its noninvasiveness; patients only need to provide blood or other bodily fluid samples without undergoing traditional tissue biopsy. This not only reduces physical discomfort and risks for patients but also enables continuous monitoring to capture dynamic changes in the disease [4–6]. However, there are challenges regarding liquid biopsy such as limitations in detection sensitivity and specificity, as well as the need for standardized analytical methods. With ongoing technological advancements and deeper research, liquid biopsy will likely become a critical tool in personalized medicine, facilitating the development of more precise and timely treatment strategies for patients.

Currently, there are numerous clinical studies on this topic, but the use of different biomarkers and various diagnostic criteria contributes to confusion among clinicians. Thus, a common approach needs to be developed to facilitate better application in clinical practice. This review first introduces several common biomarkers assessed via liquid biopsy, elaborating on their clinical significance, detection methods, research progress, etc. For the first time, this review systematically introduces the clinical effectiveness and complementarity of the combined detection of CTCs and ctDNA. Owing to the significant advantages of combined detection and the absence of relevant reviews, this part is considered a highlight.

## Main classification of biomarkers in cancer liquid biopsy

Owing to the rapid development of technology, liquid biopsy has also advanced rapidly as a method for analyzing solid tumors. New biomarkers have continuously emerged. This section includes a summary of common biomarkers and a discussion on their development history.

### Circulating tumor cells (CTCs)

CTCs are tumor cells that are separated from the original tumor or the site of metastasis and circulate in peripheral blood. CTCs were first discovered in the plasma of a cancer patient with metastasis in 1869 [7]. However, there was limited research on CTCs until Massimo Cristofanilli and others discovered in 2004 that CTC count is an independent prognostic indicator for advanced breast cancer [8]. Furthermore, in 2010, the discovery of CTCs in early-stage tumors indicated that assessment of CTC counts can also facilitate the early diagnosis of cancer [9]. It was not until recently that a consensus was reached regarding the function of CTCs in the context of breast cancer [10]. Since then, CTCs have become a topic of public interest, and many studies in this area have emerged.

### Circulating tumor DNA (ctDNA)

ctDNA refers to the DNA released into the circulatory system by tumor cells through shedding or apoptosis. It acts as a unique tumor biomarker and is an emerging potential biomarker [11, 12]. In 1948, ctDNA was first discovered in the bloodstream [13]. In 1977, Leon and colleagues reported increased cell-free DNA (cfDNA) content in the blood of tumor patients [14]. In a 1994 study, DNA with specific KRAS mutations was found in the entire bloodstream of patients with pancreatic tumors [15]. In 2015, ctDNA testing was employed for the detection of minimal residual disease (MRD) and for predicting the probability of early breast cancer reappearance, aiding in the tailoring of alternative adjuvant treatment approaches [16]. When recurrent lesions cannot be detected through imaging, the presence of ctDNA in the body can act as an indicator. A decrease in ctDNA levels can be observed when treatment is successful, whereas an increase in ctDNA levels is associated with poor treatment outcomes or tumor recurrence.

### Exosomes, extracellular vesicles (EVs) and microRNAs

In addition to CTCs and ctDNA, tumor-derived exosomes and other extracellular vesicles (EVs) are another type of circulating marker assessed in liquid biopsy samples from cancer patients. Exosomes were discovered as early as the end of the last century, but they were considered only a type of cellular metabolic waste [17]. As research has progressed, researchers have shown that exosomes might mediate communication between cancer cells [18]. Exosomes typically range in size from tens to several hundred nanometers and are a type of extracellular vesicle (EV) that can be released into bodily fluids through fusion with the plasma membrane or by active budding [19, 20]. Owing to the characteristics of exosomes and the proteins, DNA, and various RNAs they contain, they can serve as effective complements to the liquid biopsy components mentioned earlier.

In 2006, RNA was first discovered in exosomes from cell cultures [21], and subsequent studies detected tumor-derived mutations in exosomes from the blood of cancer patients [22]. This presents an opportunity to use exosomes for diagnostics. Most RNAs in exosomes have a length of less than 700 bp, which is why research on RNAs that may serve as diagnostic markers has focused on microRNAs [23–26]. According to recent research findings, the mechanisms underlying microRNA dysregulation primarily involve the amplification or deletion of microRNA genes, epigenetic dysregulation, and anomalies in transcription factor activity [27].

## Methods for detecting major biomarkers in liquid biopsy and related technical challenges

With the development of various detection methods, the technical challenges faced in biomarker extraction are also changing, evolving from the initial question of how to extract biomarkers to how to extract them with high precision and low cost. This section mainly introduces the commonly used detection methods for each biomarker, along with their respective advantages and disadvantages, while also highlighting the main issues currently faced in the extraction process.

### Methods for detecting circulating tumor cells and challenges

Various isolation strategies have been developed since the initial discovery of CTCs. Nevertheless, their detection remains challenging because of factors such as their low levels in peripheral blood (approximately 1 to 100 cells per milliliter of blood and a brief lifespan in circulation (1 to 2.5 h)) and the absence of cancer-specific markers, thus reducing their potential diagnostic value [28, 29]. Consequently, numerous platforms have been designed to address this challenge by identifying CTCs in blood samples. This analytical process involves enrichment, identification, and examination. Technologies for capturing CTCs encompass biophysical enrichment as well as strategies for positive and negative selection based on immunoreactivity. Multiple methods have been devised to extract CTCs from blood samples, with consideration of their physical characteristics (dimensions, flexibility, density, and electric charge) and physiological characteristics and the expression of various cancer markers. Racila et al. utilized ferrofluids coupled with flow cytometry (FCM) for CTC detection; for this, immunomagnetic CTC enrichment was performed using an antibody targeting epithelial cell adhesion molecule (EpCAM) [30]. The CellSearch system is a widely accepted method for CTC detection and has been approved by the FDA for detecting CTCs in colorectal cancer (CRC). However, the EpCAM bias introduced in the enriched CTC population remains a significant limitation of immunocapture methods, including CellSearch [31]. Other technologies, such as theAdnaTest, isolation by size of epithelial tumor cells (ISET), and size-dictated immunocapture chips, are employed for enrichment, detection, and separation of CTCs due to the potential ineffectiveness of immunocapture approaches caused by the downregulation of EpCAM in cells that have undergone EMT, which may lead to false-negative results [32]. Moreover, over the past decade, successful instances of combined isolation technologies have included CTC-Chip and CTCs Cluster Chip. The broader implementation of microfluidic technology for capturing rare cells among patients with tumors shows that establishment of a reliable framework for CTC detection has considerable promise in revealing important biological features of metastases in the bloodstream and for the detection and surveillance of early cancer [33, 34]. Additionally, advancements in nanotechnologies have been made to improve the precision and accuracy of CTC detection. In their review, Wenzhe Li and colleagues assessed the utilization of nanotechnology-based liquid biopsy, offering a novel outlook on tumor surveillance and therapy in clinical practice [35]. Xenotransplantation into mice increases the abundance of CTCs in a cell culture and aids in subsequent exploration [36]. In conclusion, employing various methods for CTC capture is likely imperative, which will advance future cancer studies and require validation.

### Methods for detecting circulating tumor DNA and challenges

Both normal and cancer cells release cfDNA into the blood circulation, ctDNA is a special type of cfDNA. Typically, ctDNA constitutes approximately 0.01–5% of the total cfDNA in individuals with cancer [37]. Although ctDNA has a half-life of two hours, it undergoes rapid clearance upon entering the circulation. Consequently, ctDNA serves as a valuable dynamic indicator of tumor volume and treatment response. In recent years, various ctDNA detection technologies, including highly sensitive targeted polymerase chain reaction (PCR)-based methods and next-generation sequencing (NGS) techniques, have emerged. The former encompasses digital polymerase chain reaction (dPCR) [38–40], et al., which identify mutations in predefined cancer-associated genes [41]. The former, exemplified by tagged-amplicon deep sequencing (TAm-Seq) [42–44], et al., allows for simultaneous detection of genomic sequences without the need for primary lesion sequencing. Conversely, untargeted techniques such as whole-genome sequencing (WGS) or whole-exome sequencing (WES) enable the identification of novel, clinically significant genomic changes without information on the original tumor. Generally, PCR-based methods offer the advantages of being cost-effective and rapid, requiring no specific bioinformatics expertise. However, these methods are limited in their ability to detect only a predefined set of mutations. Among PCR-based approaches, BEAMing excel in detecting rare mutations with high efficiency, although their application is constrained by the targeted DNA region [45]. While digital PCR (dPCR) remains the most widely used method for ctDNA detection, NGS is being increasingly utilized for this purpose. NGS-based methodologies leverage somatic single-nucleotide variant allele frequency (SNV VAF), copy number alteration (CNA), or DNA methylation profile data to estimate the levels of ctDNA in plasma [46, 47].

### Methods for detecting other emerging liquid biopsy markers and challenges

In recent decades, researchers have gradually explored extracellular vesicle isolation techniques. Common separation strategies rely on the physical and chemical properties of exosomes. These methods primarily include centrifugation, ultrafiltration, capture, microfluidic isolation, and various polymer-based isolation techniques commonly employed using commercial kits [48, 49]. This section separately introduces the separation methods based on the physical and chemical properties of the exosomes and describes the advantages and disadvantages of each separation method.

The most commonly used separation methods based on the physical properties of exosomes are ultracentrifugation and filtration. The former has been considered the optimal method for exosome separation [50]. This technology relies on the density and size differences between exosomes and other components, with simple operation techniques and high production purity. However, repeated operations can decrease exosome purity, and exosomes are prone to damage during centrifugation, making them unsuitable for clinical applications [51, 52]. The main filtration methods include ultrafiltration, sequential filtration, and size exclusion chromatography (SEC). Ultrafiltration is commonly used to initially screen the particle size of samples and reduce the sample volume [53]. Sequential filtration involves removing cells and cell debris first, followed by protein removal, and finally filtering extracellular vesicles on the basis of particle size [54]. With both methods, the particle size of the final product can be controlled. SEC is advantageous because it can be repeated, has lower costs, and does not damage EVs. Filtration-based EV separation techniques have been widely applied in many fields. A composite method known as sequential centrifugal ultrafiltration (SCUF) has been used to successfully isolate EVs human colon cancer cell lines [55], but the operational time remains too long for widespread clinical use.

The last three commonly used techniques for isolating EVs are more complex than the first two techniques are. Capture technology is a commonly used method for producing high-purity EVs. Magnetic beads that specifically bind to proteins on the surface of EVs can be used to separate these vesicles. Owing to its high selectivity, this method can be used to separate EVs into specific groups according to the cell of origin; thus, it is considered the best method for isolating EVs [56]. Reagent kits based on this method (such as QIAGEN) are widely used to purify RNA from EVs [57]. However, the high cost and relatively low yield currently limit the clinical applicability of this technology. Compared with the abovementioned separation methods, the sedimentation technique is more suitable for clinical research. For sedimentation techniques, polyethylene glycol (PEG) is commonly used for exosome enrichment, and then the samples are incubated overnight and then subjected to purification methods such as filtration or centrifugation [58]. However, each method, including sedimentation, has advantages and disadvantages. Owing to the extensive aggregation of exosomes, various contaminants (mainly albumin) are easily included [59], posing a challenge to clinical research. Increasing the number of filtration and purification steps can reduce such interference [60]. It is hoped that future research can truly advance sedimentation methods into clinical practice. The last method, and currently one of the more promising methods, is based on microfluidic technology. Currently, this approach relies mostly on the physical and chemical properties of EVs and continuous separation for complete integration [61]. Compared with the aforementioned methods, it can achieve efficient and cost-effective separation of EVs with speed and accuracy and is currently the most suitable method for clinical research [62]. However, it also has several disadvantages, such as the need for EVs to have unique immune recognition sites and the inherent complexity of the system itself [63]. A truly ideal EV separation method should be fast, inexpensive, and easily reproducible [64–66]. Currently, more advanced separation methods are needed to promote research on EVs.

The initial challenge in microRNA detection was determining how to detect ultrashort sequences, but with advancements in technology, this issue has been overcome. The initial detection techniques were northern blotting and RNA protection assays, both of which require an RNA content greater than 1 µg, which is particularly high for microRNAs. By the early twenty-first century, capture-probe microarrays and bead platforms had emerged as the main choices for analyzing large amounts of microRNAs within tumor cells [67–70]. Subsequently, reverse transcription quantitative polymerase chain reaction (RT‒qPCR) was developed; it enables the rapid amplification of small amounts of RNA material (> 25 pg) and has become the gold standard for validating microRNA expression [71]. The greatest challenge currently faced is no longer the issue of the short sequences of microRNA but rather the forms and sources of microRNA present in bodily fluids. The presence of microRNAs in body fluids can be roughly divided into free forms and bound forms, with the bound forms including protein-bound types, vesicle-containing types, and even those contained within immune cells and platelets [72–74]. Among them, free and protein-bound microRNAs may be released by lysed tumor cells or nontumor cells. MicroRNAs within vesicles, as described earlier, are most likely products of intercellular communication [75, 76]. The content of microRNAs in immune cells can vary significantly, as it depends on the activation status of tumor immunity [77–80]. Different separation strategies need to be developed for RNA from different sources while avoiding interference between them. To address this challenge, the most commonly used strategy is to add exogenous RNA, which often requires selecting a set of RNAs for analysis rather than a specific RNA [81]. However, the levels of microRNAs detected by this method can also be influenced by other factors (such as the degree of sodium and water retention in patients). It is hoped that a housekeeping microRNA can be identified soon to address this issue.

## The clinical applications of major biomarkers in liquid biopsy

In the field of liquid biopsy, several major biomarkers, including CTCs, ctDNA, exosomes, extracellular vesicles (EVs), and microRNAs, have been increasingly being applied in the early diagnosis and monitoring of cancer. CTCs and ctDNA were developed earlier, and many major cancer centers are conducting clinical trials with encouraging results. As emerging biomarkers, EVs have also facilitated significant breakthroughs in the early detection and diagnosis of various solid tumors [82]. Therefore, this section is divided into three parts, summarizing the clinical applications of each major biomarker.

### The clinical applications of circulating tumor cell

When minimal residual disease (MRD) is present in the body, a few tumor cells persist in the bloodstream, and these microlesions can circulate through the blood, eventually leading to recurrence. Lung cancer, as the primary contributor to cancer-related fatalities globally [83], has been the subject of a wealth of research related to CTCs. Adjuvant therapy is part of the standard treatment regimen for stage II-IIIA non-small cell lung cancer (NSCLC), and CTC testing results can influence decisions regarding adjuvant therapy. Existing evidence indicates that timely identification and assessment of tumors extend the postoperative overall survival (OS) of NSCLC patients [84]. These findings indicate that CTC detection may become a way for the diagnosis of MRD in individuals with lung cancer following surgical removal. Some studies indicate that CTCs plays a vital role in the precision treatment of lung carcinoma. Whenacquiring tissue biopsy samples is not viable, CTC detection serves as an effective method for identifying anaplastic lymphoma kinase (ALK) gene rearrangements in NSCLC patients [85]. Others argue that CTCs in fluidbiopsiesreflect tumor cell diversity better than tissue biopsies, thereby facilitating continuous tracking of disease progression. In another study, blood samples were collected every two months until disease progression from NSCLC patients with epidermal growth factor receptor(EGFR) mutations who were receiving erlotinib treatment; analysis revealed that in lung cancer patients,CTC assessmentbased on blood samples can complement EGFR mutationassessmentbased on tissue samples.

The limitations of traditional tissue biopsy are evident in CRC as well; they include invasiveness, sample limitations, and lack of repeatability for monitoring disease progression. Therefore, tissue biopsies do not comprehensively reflect tumor heterogeneity and cannot be used to dynamically monitor progression. In a prospective study conducted at a single center, 183 CRC patients without metastasis were enrolled. CTCs were identified before surgery and after surgery during the follow-up period. The results revealed that patients with CTCs2–3 years after surgery had a significantly worse prognosis than those without CTCs [86]. These findings indicate that CTC monitoring can provide insight into the long-term presence of MRD. The prolonged existence of MRD is not only a crucial factor in the recurrence of CRC but also a significant determinant of disease prognosis. The clinical importance of CTCs in the diagnosis and assessment of therapeutic effectiveness in patients with colorectal carcinoma has been preliminarily confirmed. CTCs are highly promising noninvasive prognostic biomarkers [87]. However, the diagnostic utility of CTCs for early-stage CRC patients is limited, as multiple experimental results indicate that different detection methods may yield varying positivity rates. Bork et al. reported CTC counts as low as 9% in the detection of early-stage CRC [88]. However, Sebastian Heinz and colleagues utilized Ficoll for mononuclear cell extraction, followed by CTC detection via the PCR method mentioned earlier, and achieved a detection rate of 30% [89]. Indeed, discovering a highly accurate and fast detection method is crucial for the application of CTCs as pivotal instruments in the early detection of cancer. Precision therapy refers to drug treatments tailored to individual tumor characteristics [90]. Variations in patient responses to drug therapy may stem from tumor heterogeneity, differences in gene expression, and polymorphisms. CTC detection are highly available for the early diagnosis of tumors, treatment response prediction and decision-making, prognosis assessment, and personalized therapy. CTC analysis, which is userfriendly, noninvasive, and highly reproducible, enables real-time monitoring based on the analysis of RNA, DNA, and protein molecules [91, 92]. CTC detection, a noninvasive procedure for tissue sampling, aids in the screening of early diagnostic markers for cancer [93, 94]. CTCs in the bloodstream serve as optimal target markers in biopsy samplesthat can reflect both individual and tumor heterogeneity.

The pathway mediated by estrogen receptor (ER) is the pivotal driving factor for tumor growth in ER-positive breast cancer patients. Notable research, such as that conducted by the PAOLETTI team, has facilitated the development of a multiparameter CTC-endocrine therapy index (CTC-ETI) based on CellSearch, and clinical trials to assess the clinical relevance of theCTC-ETI are ongoing [95]. Her2 gene expression status determines the subtype of breast cancer, and Her2 a key therapeutic target for breast cancer treatment. However, compared with primary tumors, distal metastatic breast cancer and CTCs in the body exhibit variable Her2 statuses [96]. The mechanism behind this phenomenon remains unclear. The Jordan team discovered that this inconsistency is due to transcriptional plasticity [97]. Relevant clinical trials, such as DETECT-III and CTC-TREAT, are ongoing. Similarly, in hormone-sensitive tumors, a similar phenomenon has been reported in the study of prostate cancer, where mutations detected in CTCs are not consistent with mutations in the primary site [98]. Prostate-specific membrane antigen (PSMA) is a transmembrane protein [99]. A phase II clinical trial reported on the use of BIND-014 together with prednisone for prostate cancer therapy and indicated that the CTCs eliminated from the bloodstream consist primarily of cells that express PSMA [100]. Therefore, research in the field of CTCs has provided new methods for tumor assessment and has significantly increased the accuracy of personalized treatment.

CTC assessment facilitatestailored treatment decision-making in cases where suitable tissue biopsy samples are unavailable [101]. Consequently, the DNA identified through CTC analysis is indicative of the development of drug resistance in cancer. Continuous monitoring of cancer treatment response through CTC analysis holds significant value and research prospects. We summarize several common solid tumors and their clinical studies related to CTCs, with the search terms being CTC and solid tumors. One study on CRC reveled that the CTC count did not help predict prognosis. More clinical and preclinical research is needed to investigate the clinical effectiveness of CTCs in solid neoplasms (Table 1).

**Table 1:** Recent summary table of clinical studies on solid tumors related to CTCs

TYPE OF TUMOR	PATIENTS(n)	ANALYSIS TIME-POINT	DETECTION METHODS	RESULTS	NCT NUMBER	REFS
NSCLC	101	At baseline	CellSearch	CTC is a novel prognostic factor for NSCLC	NCT02407327	[102]
	54	At baseline, or later, at progression	CellSearch	Survival was worse in patients with PD-L1-positive CTCs	NCT02866149	[103]
	199	At baseline, and after therapy	CellSearch Expanded cytokeratins profile (EA)	Patients with ≥ 4 CTCs had significantly shorter PFS and OS. Similar results were obtained using 5 CTCs as the cutoff value. NSCLC patients with EML4-ALK-positive CTCs exhibited shorter PFS than those with EML4-ALK-negative CTCs	NCT02407327	[104]
SCLC	75	At baseline	CellSearch	CTC counts with thresholds of 2, 15 and 50 are all significantly correlated with PFS and OS. The presence of ≥ 15 CTCs at baseline is an independent indicator of poor survival	NCT00433563	[105]
	152	At baseline	CellSearch	The median OS was 10.5 months among individuals whose baseline CTC counts were ≤ 100/7.5 mL, while it was 7.2 months for those with higher CTC counts	NCT00887159	[106]
CRC	1	At baseline	CellSearch	CTC cell line analysis can serve as a method for monitoring treatment response	NCT01596790	[107]
	25	At baseline and after therapy	CellSearch and FMSA	Using the FMSA method to isolate CTCs is more precise	NCT01722903	[108]
	1208	At baseline	CellSearch	CTC counts are unrelated to RAS, BRAF, or MSI-H molecular tumor profiles. High CTC levels and RAS mutation are associated with a poor prognosis	NCT01640405 NCT01640444	[109]
BRC	547	At baseline	CellSearch	Patients with CTCs exhibited a 12.7-fold increase in recurrence risk	NCT00433511	[110]
	165	At baseline	Maintrac	In patients where the number of CTCs increases during treatment, prognosis was worse and the risk of metastasis is higher	NCT03935802	[111]
	221	At baseline and after therapy	CellSearch	CTC can be effectively detected in the blood of HER2-positive breast cancer patients using the CTC RT-qDX method	NCT02450422	[112]
	50	At baseline	CellSearch	By detecting CTCs, the analysis efficiency of mutated genes is higher	NCT01701050	[95]
	56	At baseline and after therapy	CellSearch	CTC levels can serve as a biological parameter for advanced metastatic breast cancer	NCT01349842	[113]
PC	72	At baseline	CellSearch	CTCs have high diagnostic value for pancreatic cancer and provide guidance for treatment decision-making	NCT03032913	[114]
	79	At baseline and after two months of therapy	CellSearch	Positive CTCs are associated with poor tumor differentiation, shorter OS, and anemia	NCT00634725	[115]
	45	At baseline	CellSearch	CTC is a biomarker for pancreatic cancer patients	NCT03340844	[116]
EC	96	At baseline and after treatment	CellSearch	The detection of CTCs at any time after treatment indicates poorer DFS and OS	NCT00907543	[117]

Data sources – ClinicalTrials.gov

*Abbreviations*: *NSCLC* non-small cell lung cancer, *SCLC* small cell lung cancer, *CRC* colorectal cancer, *BRCA* breast cancer, *PC* pancreatic cancer, *EC* esophageal cancer

### The clinical applications of circulating tumor DNA

ctDNA constitutes only a small fraction (less than 1%) of the circulating free DNA in an individual [41, 118, 119]. There have been an increasing number of studies on ctDNA in the field of solid tumors, and improvements in detection technologies have increased the sensitivity of ctDNA detection. As a result, ctDNA analysis is gradually being accepted in the field of oncology. A study from the Stanford University Department of Radiation Oncology indicated that while immunotherapy for non-small cell lung cancer can yield lasting and significant effects, most sick people experience early disease progression. Therefore, early detection of tumor survival status and treatment effectiveness are crucial. An experiment utilizing immune analysis and targeted analysis of the ctDNA treatment response revealed that treatment effectiveness in lung cancer patients receiving immunotherapy can be determined by monitoring changes in ctDNA after treatment [120]. Therefore, ctDNA assessment can be used to detect early-stage cancer effectively, enabling prompt intervention and potentially preventing progression to advanced stages of the disease.

Recently, the journal Nature Cancer published the results of a phase II prospective clinical study named INSPIRE. The INSPIRE study evaluated the ability of plasma ctDNA to predict the efficacy of the antibody–drug conjugate patritumab deruxtecan. The results revealed a significant correlation between plasma ctDNA levels and treatment effectiveness, and ctDNA could predict treatment response earlier than imaging. This study highlights that ctDNA not only serves as an indicator for detecting tumor recurrence but also provides compelling evidence that ctDNA can serve as a biomarker in immunotherapy [121]. In a study on neoadjuvant therapy for breast cancer, 84 high-risk early-stage breast cancer patients were selected, and 291 plasma samples were collected from these patients. Personalized ctDNA analysis was performed to determine the connection between neoadjuvant therapy and the likelihood of a pathological complete response (pCR) and metastatic recurrence in breast cancer patients. The findings indicated that among the 84 patients, 61 (73%) exhibited ctDNAoverexpression, and the proportion of ctDNA-positive patients gradually decreased as the treatment progressed. Seventeen patients achieved a pathological complete response (pCR) after neoadjuvant therapy, and all of them had negative ctDNA results. For the remaining 43 patients who did not achieve pCR, 14% had a high likelihood of metastatic relapse, whereas the remaining 86%, despite not achieving pCR, had negative ctDNA results and demonstrated a health status similar to that ofpatients with a pCR in subsequent follow-ups [122]. The findings of this study highlight the high predictive value of ctDNA detection, providing a meaningful indication of the risk of recurrence. These findings suggest that ctDNA results can precisely predict the outlook for patients and may become one of the key indicators for evaluating treatment effectiveness and prognosis in the future. In early-stage treatment, ctDNA has displayed exceptional sensitivity in indicating treatment effectiveness, providing a precise and sensitive marker for clinical interventions. This enables doctors to promptly understand a patient's responsiveness to therapy, allowing for timely adjustments in treatment plans for those less responsive to the current interventions.

For postoperative patients, there is often a longer survival period, making the monitoring of recurrence and the choice of adjuvant therapy extremely important. In an experiment analyzing individuals diagnosed with stage I to III CRC, 149 patients, with an average age of 67.9 years, including 74 (56.9%) males, were enrolled. Among the 108 patients, 14 patients had detectable ctDNA postoperatively, indicating recurrence; thus, ctDNA assessment was able to detect recurrence in 87.5% of the 16 patients who actually presented with recurrence. At postoperative day 30, patients with ctDNA overexpression had a sevenfold greater likelihood of relapse than patients with low ctDNA expression. Among the 10 ctDNA-positive patients who underwent neoadjuvant chemotherapy, 3 patients (30%) became negative for ctDNA and showed no signs of recurrence, whereas the remaining 7 patients who were still positive for ctDNA presented with recurrence. Continuous ctDNA analysis revealed that CRC recurrence can be detected 16.5 months earlier with ctDNA monitoring than with conventional imaging studies [123]. These findings demonstrate that MRD assessed via ctDNA detection plays a role in assessing risk, evaluating adjuvant treatment response, and identifying early tumor recurrence in patients. ctDNA not only is useful for evaluating recurrence risk and treatment benefits in stage III colon cancer patients but also has been studied in 230 stage II colon cancer patients. Among the 178 patients who did not undergo additional chemotherapy, 14 patients (7.9%) were ctDNA-positive postoperatively, and 11 of these patients (78.6%) presented with recurrence during follow-up. Among the remaining 164 patients with negative postoperative ctDNA results, only 16 patients (9.8%) presented with recurrence [124]. The relapse frequency in ctDNA-negative patients is lower than that in ctDNA-positive patients. These findings indicate that assessing the presence of ctDNA postoperatively can be useful for evaluating the likelihood of relapse in patients. These findings underscore the advantages of MRD assessment based on ctDNA in tumor treatment. Owing to the different characteristics of various cancers, early-stage CRC is slightly more common than other early-stage solid tumors, and the number of patients eligible for surgery is somewhat greater. We searched for literature related to the relationship between ctDNA and postoperative tumor treatment (with the search terms "surgery" and "ctDNA") and compiled the information into a table. The table includes the tumor type, number of enrolled patients, measurement time points, testing methods, and results (Table 2).

**Table 2:** Summary of recent clinical studies on solid tumors related to ctDNA

TYPE OF TUMOR	PATIENTS(n)	ANALYSIS TIME-POINT	DETECTION METHODS	RESULTS	NCT NUMBER	REFS
NSCLC	46	At baseline	Oncomine Tumor Mutation Load Assay	There are significant correlations between low ctDNA levels before and after treatment and PFS and OS	NCT03081689	[125]
	45	At baseline, during neoadjuvant treatment	QIAamp Circulating Nucleic Acid Kit	Patients with undetectable ctDNA have a higher pCR rate and a higher 18-month event-free survival (EFS) rate	NCT04015778	[126]
BRCA	161	Three-monthly blood sampling to 12 months	digital PCR	ctDNA-positive patients have a higher metastasis rate	NCT03145961	[127]
CRC	192	At baseline, after therapy	QIAamp Circulating Nucleic Acid Kit	Compared to single biomarkers, combined ctDNA detection has greater clinical accuracy in predicting treatment outcomes for mCRC patients	NCT01212510	[128]
	513	At baseline	VersaBio Human Genomic DNA FastPrep Kit	Methylation detection based on ctDNA has the potential to become an early diagnostic method for CRC	NCT05508503	[129]
UC	581	At baseline	multiplex PCR assay	ctDNA-positive patients have a higher risk of recurrence	NCT02450331	[130]
	95	At baseline, after therapy	Ultradeep sequencing	ctDNA is crucial for prognostic indication; no recurrence was observed in ctDNA-negative patients in the study	NCT02662309	[131]
EGJC	42	At baseline, before surgical resection, after surgical resection	Ultrasensitive targeted next-generation sequencing (NGS)	Patients with undetectable ctDNA at the postoperative time point had a longer RFS than those with detectable ctDNA	NCT03044613	[132]

Data sources – ClinicalTrials.gov

*Abbreviations*: *UC* urothelial cancer, *EGJC* esophageal/gastroesophageal junction cancer

### The clinical applications of other emerging liquid biopsy markers

Compared with those of CTCs and ctDNA, the clinical applications of exosomes are more extensive. This section elaborates on the clinical applications of exosomes from both diagnostic and therapeutic perspectives and discusses the clinical applications of microRNAs. Because exosomes are a type of EVs and their roles in the field of tumors are highly overlapping, they are not discussed separately here.

The role of exosomes in cancer diagnosis can be summarized in two points. First, unlike ctDNA, which consists of small fragments of DNA released into the circulation from dying cells through apoptosis, exosomes are continuously released by living cells. This indicates that exosomes provide information about nondead tumor cells and can significantly increase the chances of early lesion detection. Second, multiple studies have demonstrated that combined analysis is more beneficial than the analysis of a single biomarker [133–135]. Owing to the unique composition of exosomes, these vesicles contain many proteins, DNA, RNA, oligosaccharides, and metabolites, making them ideal samples for combined analysis. The role of exosomes in cancer treatment cannot be ignored. Owing to their function in intercellular communication, exosomes have been confirmed to serve as carriers for cancer therapeutic drugs [136]. Compared with commonly used drug carriers (metallic nanomaterials, liposomal nanomaterials), exosomes, as normal secretory products of cells, have significantly higher bioavailability and much lower cytotoxicity to nontarget cells [137]. It has been confirmed that target cells have a strong endocytic effect on specific exosomes [138]. Research by Kim et al. demonstrated that, compared with the use of liposomes as drug carriers, the use of exosomes derived from macrophages as drug carriers significantly increased drug uptake in mouse lung tumors [139, 140]. Moreover, drugs encapsulated in exosomes exhibit a 50-fold greater cytotoxic effect against resistant cells than free drugs, which can be attributed to the cellular uptake of exosomes. Similarly, in animal experiments, drugs encapsulated in exosomes not only inhibited tumor growth but also had significantly fewer side effects than free drugs did [141, 142].

Recent studies have indicated that microRNAs can significantly enhance liquid biopsy approaches [143, 144]. Specifically, miR-210 has been identified as an independent prognostic factor for early-stage breast cancer [145, 146], and the upregulation of miR-21 can impact the clinical stage of cancer, lymph node metastasis, and patient survival rates [147, 148]. Furthermore, the downregulation of miR-126 and miR-335 is associated with the regression of metastatic lesions in breast cancer patients [149]. Recent studies have also suggested that microRNA expression is increased in various solid tumors, including prostate cancer [150, 151] and lung cancer [152]. A study proposed a diagnostic tool that aids in the diagnosis of bladder cancer through the combination of microRNA analysis and surface-enhanced Raman scattering (SERS) of urine liquid biopsy samples. One study demonstrated that urinary microRNAs analysis and SERS analysis can predict outcomes better than either analysis type alone [153]. In the PROSPECT-R trial, large-scale microRNA analysis revealed MIR652-3p as a biomarker indicating the benefit of regorafenib treatment in advanced CRC, and these results were confirmed in a control group comprising 100 patients [154, 155]. These results indicate that microRNA-based liquid biopsy may serve as an innovative research field for identifying biomarkers in various solid tumors.

## The application of liquid biopsy based on the combined detection of CTCs and ctDNA in cancer

**Fig. 2:**
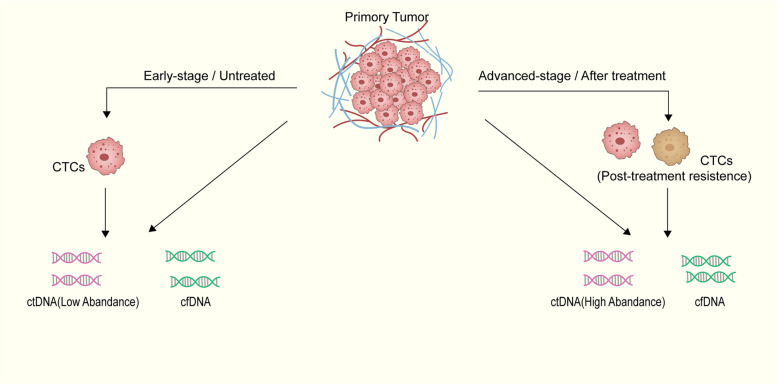
Summary of the characteristics of CTCs and ctDNA at different stages of cancer with an emphasis on their complementarity in the field of liquid biopsy. In the process of tumor occurrence and development, tumor cells and their surrounding environment are constantly changing. The differences between early-stage tumors and late-stage tumors, as well as between tumors before and after the acquisition of resistance, can be reflected at the molecular level. The characteristics of CTCs and ctDNA at different stages of the tumor are listed to illustrate the complementarity between the two

As stated above, liquid biopsy has facilitated significant advancements in cancer diagnosis, treatment monitoring, and other areas. However, the detection of single biomarkers has limitations; for example, the CELLSEARCH® CTC test is the first and only clinically validated, Food and Drug Administration (FDA)-approved blood test for the quantification of CTCs. For this test, 5 CTCs/7.5 mL whole blood is used as the cutoff. In a meta-analysis involving 2239 breast cancer patients from 21 studies, when the CTC count was less than 5, the risk of death for patients increased significantly compared to patients with the CTCs count of 0. Although the mortality risk was not as high as when the count was above 5, it still indicated a poor prognosis [156]. During the occurrence and development of tumors, both the internal and external microenvironments of tumor cells change, leading to alterations in the chemical properties of the tumor, including but not limited to changes in genetic mutations and the degree of invasiveness. Owing to the different mechanisms of formation of CTCs and ctDNA, the information they carry at different stages of the tumor also differs. Additionally, the changes in tumors within the tumor lesions are not uniform; for example, resistant cells may be mixed with nonresistant cells. Combined detection may be the key to addressing this series of issues. For convenience, the information contained in CTCs and ctDNA at different stages of cancer development is summarized in a figure (Fig. 2). This section mainly introduces the clinical effectiveness and complementarity of combined CTC and ctDNA testing.

### The clinical significance of CTC and ctDNA detection for tumor prognosis assessment and dynamic monitoring

CTCs can be released into the blood during the initial phases of tumor formation, where they participate in the distant dissemination and metastasis of tumor cells via the EMT process. On the other hand, ctDNA consists of DNA pieces carrying tumor-specific information circulating in the blood. It mainly arises from necrotic or programmed death oftumor cells or CTCs [157, 158]. CTCs, as complete tumor cells, enable single-cell level visualization for MRD monitoring at the cellular level. On the other hand, detection of plasma ctDNA provides a molecular biology perspective to reflect the presence of MRD [159, 160].

Through CTC and ctDNA detection, a comprehensive and dynamic overview of the tumor can be obtained, allowing for more effective monitoring of MRD [161]. CTC subtyping and ctDNA detection, when used in cancer diagnosis or monitoring after treatment, can lead to the early discovery of tumor recurrence, often 6–9 months ahead of detection with traditional clinical indicators or imaging [162]. A study on liver cancer indicated that, compared with individual CTC or ctDNA detection, the combined detection of CTCs and ctDNA can more accurately identify the existence of postoperative MRD in liver cancer patients and achieve more precise recurrence predictions, with a specificity of 81.6%. Compared with liver cancer patients with either CTC or ctDNA positivity (CTC + /ctDNA +) or double negativity (CTC-/ctDNA-), liver cancer patients with double positivity for CTCs and ctDNA (CTC + /ctDNA +) had significantly shorter recurrence-free survival [163]. The AZD9496 Oral SERD phase I trial was the first study to report baseline CTC and ctDNA test results simultaneously; the correlative results were obtained by detecting CTCs to identify ER-positive status and using ctDNA analysis to detect resistance-conferring mutations [164]. The combination of both methods represents a complementary approach for comprehensive monitoring of tumor microchanges. An investigation of the importance of a joint evaluation of CTCs and ctDNA revealed that both total cfDNA levels and CTCs were independently and collectively linked to PFS and OS in advanced breast cancer patients [165]. Another comparable study similarly indicated that liquid biopsy provides prognostic insights [166]. In the preplanned secondary analysis of the BRE12-158 randomized clinical trial, CTCs and ctDNA were used as combined MRD indicators to assess the risk of metastasis in individuals with early triple-negative breast cancer undergoing neoadjuvant treatment. MRD detection sensitivity, which was based on both CTC and ctDNA positivity, reached 90% (with individual sensitivities of 62% and 79% when these indicators were used separately). At 24 months, the probability of distant disease-free survival (DDFS) for patients with CTC and ctDNA overexpression was 52%, whereas it was 89% for those with negative results for both. A similar trend was also observed for DFS [167]. Similarly, in the randomized trial SWOG S1222, although the CTC count alone may have prognostic value, the combination of CTC and ctDNA analysis more clearly reflected the changes in tumors within the patient's body [168]. Interestingly, when CTCs and ctDNA first entered the public eye, the mechanisms behind both were not yet clear. As a result, few studies have compared the quantities of CTCs and ctDNA in the same patient. A study published in 2008 in the New England Journal of Medicine indicated that the genetic profiling of CTCs appeared to have higher sensitivity than the analysis of free plasma DNA. The reason for this is that free plasma DNA includes not only DNA released from apoptotic tumor cells but also DNA released from normal cells undergoing apoptosis [169]. However, in 2013, another study published in the same journal indicated that ctDNA exhibited a greater dynamic range than did CA-153 or CTCs, ctDNA levels were more strongly correlated with tumor burden than the other markers [170]. This can be attributed to the fact that the patients in the 2013 study were diagnosed with metastatic breast cancer at a later stage, where tumor cell necrosis was more prevalent, resulting in higher levels of plasma ctDNA. Thus, the combined detection of CTCs and ctDNA better reflects the dynamic changes in the tumor, whether in the early or late stages of the disease.

### The clinical feasibility of the combined application of CTCs and ctDNA in mutation detection

Many studies have confirmed that CTC and ctDNA assessments can provide complementary genetic information. A study utilizing a blood-based molecular test with high sensitivity and specificity to identify ESR1 alterations in CTCs and ctDNA from individuals with breast cancer revealed discordance in ESR1 mutation results. Specifically, no ESR1 alterations were detected in ctDNA at the initial stage, but during the follow-up period, the D538G alteration was detected in five consecutive ctDNA samples from the same individual. In contrast, in terms of the EpCAM-isolated CTC proportion, only Y537C mutations were initially identified in one individual's sample [171]. Comparative research examining PIK3CA mutations in CTCs and matched ctDNA using identical molecular tests and blood samples has demonstrated that PIK3CA hotspot mutations are frequently present in CTCs obtained from CellSearch® cartridges and matched plasma ctDNA in both early- and advanced-stage breast cancer. The study also revealed that the identification of PIK3CA hotspot mutations and concordance between plasma ctDNA and CTC assessments are more pronounced in patients with stage IV disease. Furthermore, the mutational status of PIK3CA significantly changes following treatment, and the detection of PIK3CA mutations in CTCs and plasma ctDNA offers additional insights [172]. The use of crystal dPCR to detect EGFR mutations in plasma cfDNA and corresponding CTCs from NSCLC patients before and after osimertinib treatment revealed complementary findings, but the results differed between CTC and ctDNA assessments [173]. The inconsistency between mutation status in CTC and ctDNA assessments can be attributed to the fact that, as treatment progresses, resistance mechanisms develop in most solid tumors through the occurrence of resistance-conferring mutations (especially in tumors such as breast cancer). This is a progressive process, and at the time points when CTCs and ctDNA are detected, the extracted cells or DNA may not have necessarily acquired resistance-conferring mutations. The detection of both these markers can reduce the likelihood of such "misdiagnosis". In a recent preliminary study involving a direct comparison of paired CTC and ctDNA samples from only 16 individuals with advanced urothelial cancer, it was similarly demonstrated that CTCs and ctDNA offer complementary insights [174].

**Fig. 3:**
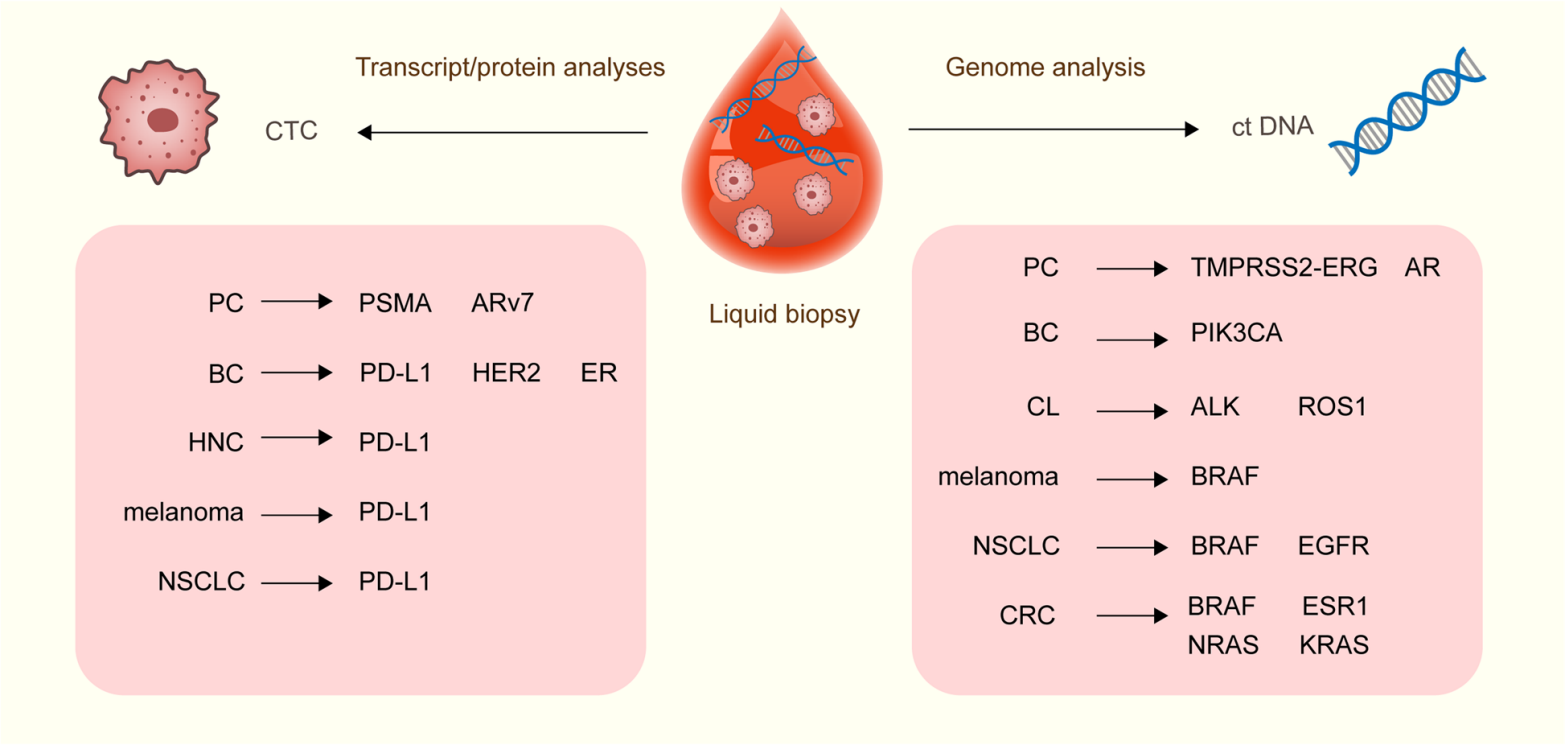
Summary of tumor treatment targets and resistance mutations based on CTC and ctDNA detection. The tumor types include non-small cell lung cancer (EGFR, ROS1, ALK), colorectal cancer (NRAS), breast cancer (PIK3CA, ESR1), prostate cancer (ARv7), and melanoma (BRAF); PD-L1 is also targeted in various tumors. Through the analysis of ctDNA, it is possible to detect drug resistance-conferring mutations that occur during the treatment process, while CTC analysis can provide insights into changes in protein expression within tumor cells that can be used to infer whether related mutations occur in resistance genes. CTCs and ctDNA are accurate markers for liquid biopsy and provide complementary information

In summary, the aforementioned studies demonstrate that CTCs and ctDNA assessments are complementary in the detection of mutations in individuals with cancer. A summary diagram of tumor treatment targets and resistance-conferring mutations based on CTC and ctDNA detection is provided to help readers better understand the clinical significance of their combined assessment (Fig. 3). The combined assessment of CTCs and ctDNA may improve the precision of MRD detection in patients with malignant tumors, achieving a "needle in a haystack" detection strategy.

## The application of machine learning in liquid biopsy data analysis

**Fig. 4:**
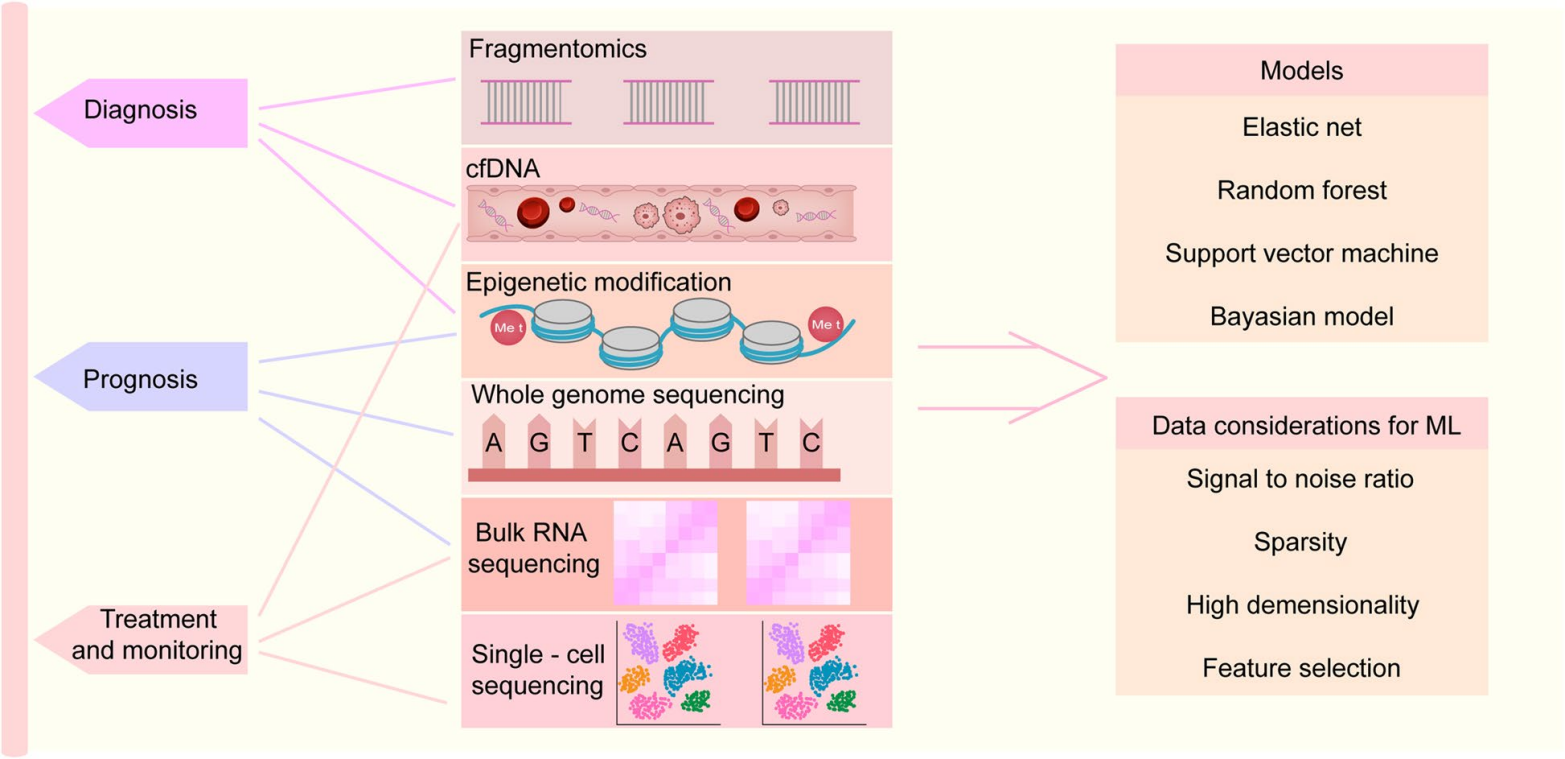
A simple summary diagram on the applications of machine learning in the analysis of liquid biopsy data. It shows what datasets are used for machine learning in the contexts of cancer diagnosis, prognosis evaluation, and treatment effect monitoring and lists common types of machine learning models and considerations for data analysis

In recent years, machine learning has developed rapidly in the field of oncology [175]. The occurrence and development of tumors are often accompanied by genetic mutations, which not only lead to complex biological behaviors of tumors but also lead to the accumulation of a large amount of clinical data. Thus, machine learning has great potential value in the field of oncology [176]. Through machine learning, image data and sequencing data can be used to identify patterns, and the emerging field of liquid biopsy, particularly ctDNA analysis, provides rich datasets for machine learning. This section mainly outlines the application of machine learning models in the field of liquid biopsy, especially for diagnosis, prognosis prediction, and treatment response monitoring in solid tumors. For convenience, a simple summary diagram on the applications of machine learning in the analysis of liquid biopsy data is provided (Fig. 4).

### Diagnosis

As mentioned earlier, liquid biopsy can be used to assess the presence of cancer by detecting genetic mutations in samples. However, a single predictive indicator has unavoidable limitations; for example, mutation burden may have strong sensitivity and/or strong specificity in cancer detection. Machine learning models can classify and analyze the detected mutations through training to identify mutations with the most clinical value. For example, F. Mouliere and others analyzed the probability of the presence of ctDNA in the plasma samples of lung cancer patients using models such as logistic regression and elastic net [177]. However, determining the tissue origin of ctDNA is a challenge. Fortunately, specific DNA methylation fragments of CpG islands can be assessed to address this issue. As the earliest abnormalities that occur during cancer development, they serve as reliable markers for determining the tissue origin of ctDNA. Furthermore, machine learning has been applied to analyze whole-genome sequencing data of cfDNA and various other data types; such studies have revealed that whole-genome methylation sequences have the highest predictive value for distinguishing the tissue origin of ctDNA [178]. However, this does not prove that short sequences of cfDNA are useless in the field of machine learning. Research has confirmed that short sequences can significantly increase the diagnostic capabilities of machine learning models [179]. This may be because short sequences carry various mechanistic information; for example, Esfahani used short-sequence ctDNA to assess the response of NSCLC patients to immunotherapy [180].

### Prognosis

In terms of cancer prognosis, the greatest role of machine learning is in determining the primary organ of metastatic tumors. This is also an important factor in assessing tumor prognosis, as clinicians can obtain key information such as the differentiation status of the tumor from it. Researchers developed the random forest model for this purpose; the main observation indicator in this context is the expression characteristics of genes [181]. Tang et al. trained a random forest model using sequencing data from 17 types of solid tumors to predict the origins of the tumors [182]. Similarly, Nguyen et al. trained a random forest model using data for primary and metastatic tumors from 6756 whole-genome sequencing samples to predict the origin of the tumors [183]. However, these applications are not based on liquid biopsy samples but rather on traditional biopsy samples. With the expansion of liquid biopsy sequencing data, machine learning will likely rapidly develop in this field.

### Treatment and monitoring

Clinical treatment decision-making for cancer patients relies entirely on treatment guidelines and clinical trial results. However, compared with machine learning, traditional clinical trials face the limitation of small sample sizes. It is unclear whether the selected treatments are optimal for the vast majority of different types of patients. Integrating and analyzing the vast amount of sequencing data and complex clinical information of cancer patients through machine learning may be an effective solution to this problem. For example, the continuous individualized risk index (CIRI), which can be used to analyze small datasets and identify patterns amidst a large amount of uncertainty, has been used to simulate the dynamic changes in ctDNA in the bodies of cancer patients undergoing different treatments [184]. It can also be used to assess patient treatment response; for example, it was applied to simulate changes in the ctDNA of non-small cell lung cancer patients undergoing immunotherapy [185]. In the field of liquid biopsy, single-cell transcriptomics can be applied to analyze CTCs to determine their cellular components, and machine learning models can be subsequently used to analyze these components to predict drug sensitivity and the likelihood of resistance [186]. In summary, machine learning has great potential in the field of liquid biopsy. The use of machine learning to analyze clinical data and make predictions may significantly improve the survival rates and quality of life of cancer patients in the near future.

## Future perspectives and conclusions

In recent years, the emerging field of liquid biopsy research has revealed new strategies for cancer diagnosis and treatment, facilitating the development of personalized therapies for tumors. The detection accuracy of CTCs and ctDNA, the most commonly used biomarkers in liquid biopsy, has consistently been a key topic in the field of liquid biopsy [187]. The accuracy of the results from different detection technologies depends on the concentrations of these two biomarkers. The standardization of analytical procedures determines whether widespread clinical application can be achieved. Currently, both BloodPac in the United States and Cancer-ID in Europe are dedicated to establishing standardized detection protocols [16, 188]. The biological processes of CTC and ctDNA formation remain major challenges. There is no direct evidence to suggest that the release of CTCs from tumor tissue is a random process. Experimental models indicate that tumor cells are already prepared for metastasis before shedding in the form of CTCs [189, 190]. The release of ctDNA typically occurs in two scenarios: during cellular apoptosis or through release via exosomes [191]. The exosomal release of ctDNA may be regulated by the tumor, and any intervention in this process through therapeutic measures may significantly impact tumor prognosis [192]. Therefore, further studies on these topics are needed.

However, each approach has some limitations. Indeed, numerous technical challenges continue to impede the effective translation of liquid biopsy biomarkers into clinical practice. Primarily, there is a lack of standardized methodologies for isolation, enrichment, or detection. Consequently, the utilization of different technologies or assays for detecting CTCs or ctDNA may result in varying sensitivities and specificities [193, 194]. Owing to the rapid development in the field of liquid biopsy, although issues related to detection accuracy still exist, they are no longer the primary concern. The common limitations of ongoing clinical studies include small sample sizes, the lack of control groups while patients are undergoing treatment, and the fact that each clinical study often only evaluates the most commonly mutated genes. Finally, there is an urgent need for larger, longer-term, multicenter studies to facilitate the clinical implementation of liquid biopsies, including clinical trials.

There is also potential for other blood components, such as exosomes and tumor-educated platelets (TEPs), to serve as biomarkers for liquid biopsy. Exosomes play diverse roles throughout the entire process of tumor occurrence and development; for example, they play roles in EMT, neovascularization in tumors, remodeling of the extracellular matrix, metastasis to specific organs, and evasion of immune surveillance. Additionally, exosomes can contribute to drug resistance in cancer. Since they are easier to isolate than CTCs and cfDNA, exosomes are increasingly becoming a focus of studies for early cancer diagnosis. However, challenges persist in their clinical application, including low targeting efficiency and susceptibility to immune clearance. Moreover, the process of isolating and purifying exosomes is time-consuming and requires significant resources. Hence, further research is needed to address these issues and develop more effective clinical application strategies. On the other hand, tumor-educated platelets (TEPs) offer advantages such as their abundance in blood, ease of isolation, high-quality RNA content, and responsiveness to external signals [195–198]. The integration of TEP RNA analysis with other biomarkers, such as exosomes, cfDNA, and CTCs, holds promise for early tumor diagnosis and noninvasive disease surveillance. The potential of fluid biopsies as predictive biomarkers for immunotherapy represents a next frontier. The tumor mutational burden (TMB) in cfDNA and immune checkpoint protein expression in CTCs are crucial factors in the response to tumor immunotherapy. Despite the current lack of a comprehensive understanding of the molecular mechanisms involved, promising data suggest that liquid biopsy has the potential to facilitate personalized clinical management. Recent technological advances also indicate that liquid biopsy may be feasibly applied to benefit cancer patients. Liquid biopsy may improve the care of patients receiving immune checkpoint inhibitors (ICIs). However, further research, particularly within the framework of clinical trials, is essential to validate this hypothesis.

It seems unlikely that tumor biopsies will be replaced by liquid biopsies, but advancements in blood-based tests suggest a growing utilization of these methods in cancer for early diagnosis, surveillance after surgery, assessment of treatment responses, and identification of treatment resistance. In essence, liquid biopsy represents an integral component of personalized medicine and is anticipated to become a clinical practice in the foreseeable future.

## Data Availability

Not applicable.

## Abbreviations

FCM: Flow cytometry
EpCAM: Epithelial cell adhesion molecule
FISH: Fluorescence-assisted in situ hybridization
FAST: Fiber-optic array scanning technology
EMT: Epithelial-to-mesenchymal transition
ALK: Anaplastic lymphoma kinase
NSCLC: Non-small cell lung cancer
ER: Estrogen receptors
CTC-ETI: CTC-endocrine therapy index
SCLC: Small cell lung cancer
BRCA: Breast cancer
CRC: Colorectal cancer
DDFS: Distant disease-free survival
BCSS: Breast cancer-specific survival
DFS: Disease-free survival
EOC: Epithelial ovarian cancer
RP: Radical prostatectomy
PC: Pancreatic cancer
EC: Esophageal cancer
PCa: Prostate cancer
NGS: Next-generation sequencing
PCR: Polymerase chain reaction
dPCR: Digital polymerase chain reaction
ARMS: Allele-specific amplification refractory mutation system
AS‒PCR: Allele-specific polymerase chain reaction
ddPCR: Droplet digital polymerase chain reaction
BEAM: Bead emulsification amplification and magnetics
TAm-Seq: Tagged-amplicon deep sequencing
ISET: Isolation by size of epithelial tumor cells
EPISPOT: Enzyme-linked immunosorbent spot assay
Safe-SeqS: Safe-Sequencing System
CAPP-Seq: Cancer personalized profiling by deep sequencing
WGS: Whole-genome sequencing
WES: Whole-exome sequencing
SNV VAFs: Somatic single-nucleotide variant allele frequencies
CNAs: Copy number aberrations
EFS: Event-free survival
RFI: Recurrence-free interval
EGJC: Esophageal/gastroesophageal junction cancer
HCC: Hepatocellular carcinoma
UC: Urothelial cancer
